# Evaluating the Protective Role of Intranasally Administered Avian-Derived IgY Against SARS-CoV-2 in Syrian Hamster Models

**DOI:** 10.3390/vaccines12121422

**Published:** 2024-12-17

**Authors:** Mónika Madai, Dániel Hanna, Roland Hetényi, Fanni Földes, Zsófia Lanszki, Brigitta Zana, Balázs Somogyi, Henrietta Papp, Anett Kuczmog, Orsolya Faragó-Sipos, Csaba Nemes, Vilmos Palya, Dávid Géza Horváth, Gyula Balka, Krisztián Bányai, Xinkai Jia, Péter Balogh, Pál Bajnóczi

**Affiliations:** 1 National Laboratory of Virology, Szentágothai Research Centre, University of Pécs, 7624 Pécs, Hungary; daniel.hanna@aok.pte.hu (D.H.); roland.hetenyi@aok.pte.hu (R.H.); fanni4444@gmail.com (F.F.); lanszki.zsofia@pte.hu (Z.L.); zana.brigitta@pte.hu (B.Z.); somogyi.balazs@pte.hu (B.S.); phencsi@gmail.com (H.P.); kuczmog.anett@pte.hu (A.K.); bkrota@hotmail.com (K.B.); 2 RoLink Biotechnology Kft., 7623 Pécs, Hungary; 3 Institute of Biology, Faculty of Sciences, University of Pécs, 7624 Pécs, Hungary; 4 Prophyl Kft., 7700 Mohács, Hungary; osipos@prophyl.hu (O.F.-S.); csnemes@prophyl.hu (C.N.); vilmos.palya@gmail.com (V.P.); 5 Department of Pathology, University of Veterinary Medicine, 1078 Budapest, Hungary; horvath.david.geza@univet.hu (D.G.H.); balka.gyula@univet.hu (G.B.); 6 National Laboratory of Infectious Animal Diseases, Antimicrobial Resistance, Veterinary Public Health and Food Chain Safety, University of Veterinary Medicine, 1078 Budapest, Hungary; 7 Department of Pharmacology and Toxicology, University of Veterinary Medicine, 1078 Budapest, Hungary; 8 Department of Immunology and Biotechnology, Medical School, University of Pécs, 7624 Pécs, Hungary; jia.xinkai@pte.hu (X.J.); balogh.peter@pte.hu (P.B.)

**Keywords:** IgY antibodies, SARS-CoV-2, COVID-19 prophylaxis, Syrian golden hamster, viral neutralization

## Abstract

Background/Objectives: The ongoing COVID-19 pandemic has underscored the need for alternative prophylactic measures, particularly for populations for whom vaccines may not be effective or accessible. This study aims to evaluate the efficacy of intranasally administered IgY antibodies derived from hen egg yolks as a protective agent against SARS-CoV-2 infection in Syrian golden hamsters, a well-established animal model for COVID-19. Methods: Hens were immunized with the spike protein of SARS-CoV-2 to generate IgY antibodies. These antibodies were extracted from the egg yolks, purified, and their neutralizing activity was tested in vitro. Syrian golden hamsters were then treated with the IgY antibodies before being challenged with SARS-CoV-2. Viral loads were quantified using droplet digital PCR (ddPCR), and lung pathology was assessed through histopathological analysis. Results: The in vitro assays showed that IgY effectively neutralized SARS-CoV-2. In the in vivo hamster model, IgY treatment led to a significant reduction in viral loads and a marked decrease in lung consolidation and inflammation compared to the positive control group. Histopathological findings further supported the protective role of IgY in reducing lung damage caused by SARS-CoV-2. Conclusions: The results demonstrate that IgY antibodies exhibit strong antiviral activity and can significantly reduce SARS-CoV-2 viral loads and associated lung pathology in hamsters. These findings suggest that IgY could be a viable prophylactic option for preventing SARS-CoV-2 infection, particularly for individuals who cannot receive or respond to vaccines. Further studies are warranted to optimize dosage and explore the long-term efficacy of IgY antibodies.

## 1. Introduction

In late 2019, initial cases of pneumonia of unknown origin were reported in Wuhan, China. These cases were later attributed to a novel coronavirus, subsequently named SARS-CoV-2 [1]. The disease caused by this virus was named COVID-19, reflecting its relationship to the previously known SARS coronavirus. This novel virus spread rapidly worldwide, leading the World Health Organization (WHO) to declare it a Public Health Emergency of International Concern on 30 January 2020, and subsequently a pandemic on 11 March 2020 [2,3]. As COVID-19 proliferated globally, it not only posed significant health challenges but also triggered widespread social and economic disruption, underscoring the need for effective control and prevention measures.

SARS-CoV-2, part of the *Sarbecovirus* subgroup within the *Betacoronavirus* genus alongside SARS-CoV and MERS-CoV, uses the angiotensin-converting enzyme 2 (ACE2) receptor to enter cells, with the spike (S) protein playing a crucial role in this process [4]. Comprising two subunits, S1 and S2, the S1 subunit’s receptor-binding domain (RBD) binds to ACE2, while the S2 subunit facilitates membrane fusion [5]. This precise understanding of the interaction between the cellular receptor and viral receptor-binding antigen provided the basis for the formulation of numerous disease control and prevention strategies.

Although the approval of various vaccines [6] marked a significant milestone in combating the disease, the production of effective vaccines is both time-consuming and costly [7]. Furthermore, the ongoing viral evolution may lead to mutations that compromise the vaccines’ effectiveness [8]. Immunocompromised patients exhibit lower seroconversion rates [9], and vaccine hesitancy, coupled with limited access, poses challenges, particularly in less developed regions [10,11]. Hence, there remains a constant need for alternative methods to mitigate the spread of COVID-19 in the general population or among individuals with specific needs.

In cases where effective vaccination is impractical or the generated immune response is suboptimal (e.g., in the elderly or immunocompromised patients), passive immunotherapy emerges as a viable alternative for infection prevention. This therapeutic approach has been employed in treating COVID-19 patients, utilizing specific IgG antibodies derived from the plasma of recovered individuals to induce clinical improvement [12]. However, challenges such as limited IgG availability and potential side effects persist [13]. A potential avenue for passive immunotherapy involves the neutralization of the virus at the cellular entry point, and specific IgY antibodies may offer a promising solution.

Derived from egg yolk, IgY serves as a homolog to mammalian IgG, demonstrating efficacy in various respiratory and digestive diseases in both humans and animals [14,15,16,17]. The production of IgY antibodies is more hygienic, comfortable, and cost-effective compared to traditional methods of IgG collection. Importantly, IgY production aligns with the principles of animal welfare (three Rs: reduction, refinement, and replacement). Notably, immunocomplexes containing IgY do not activate the human complement system and thus are incapable of triggering antibody-dependent enhancement (ADE) reactions, as IgY cannot bind to human Fc receptors. Additionally, purified IgY antibodies exhibit remarkable stability, maintaining their titer for years when stored at 4 °C [18].

In our experiments, we developed specific IgY antibodies against SARS-CoV-2 through the immunization of SPF-laying hens. We conducted analyses to evaluate the purity and specific virus neutralization titer of these egg yolk antibodies. Our focus was not on determining the minimal effective dose, but rather on the robust examination of the potential of IgY, which is typically absent in mammals, as a viable means to mitigate the consequence of infection. This investigation aimed to assess IgY’s efficacy as a medication option in mammals, exemplified by the Syrian hamster model.

## 2. Materials and Methods

### 2.1. Antigen Preparations

Selection of Antigens and Adjuvants: For vaccine development, three distinct antigens—spike protein (S), spike protein subunit 1 (S1), and the receptor-binding domain (RBD)—were selected. These antigens were utilized in two dosages: 1 µg and 10 µg. Accompanying adjuvants included TiterMax Gold, Montanide, and a combination of Montanide with CpG oligonucleotide. All antigens were sourced as recombinant proteins with a HIS-tag from HEK 293 cells (Acrobiosystem, Newark, DE, USA, Cat. Nos. S protein—SPNC52H3; S1—S1NC52H4; RBD—SPDC52H5).

Adjuvant Formulation: Two water-in-oil-type adjuvants were employed: TiterMax (Sigma Aldrich, St. Louis, MO, USA, Cat. No. T2684) and MontanideTM ISA 71R VG (Seppic GmbH, Cologne, Germany). To potentiate the immunogenic response, Montanide was used alone and in combination with CpG oligonucleotide (sequence: 5′CTAGTTCGTCGAAGTCGTTTTGGGGGGT-3′).

Vaccine Preparations and Immunization: In total, 18 different vaccine formulations were prepared, combining the spike antigen variations with different adjuvant types. These formulations were systematically utilized in chicken immunization studies to evaluate their efficacy (Table 1).

### 2.2. Immunization of Laying Hens

Hen Housing and Grouping: In preparatory experiments, 180 sixteen-week-old Babcock Specific Pathogen-Free (SPF) laying hens were housed in a Filtered Air Positive Pressure (FAPP) environment. Each bird received individual markings for identification. The hens were then divided into 18 groups, with each group comprising 10 hens housed in separate laying cages. (Ethical Committee Approval: Baranya County Government Office Ref. No. BAI/35/56–92/2017).

Acclimatization and Immunization Schedule: A two-week acclimatization period was observed before the immunization protocol was initiated. The hens received their first subcutaneous vaccination under the neck skin on Day 0 (D0). This was followed by additional immunizations on Day 35 (D35), Day 49 (D49), and Day 125 (D125).

Antibody Response Assessment and Egg Collection: The virus-specific antibody response induced by the vaccine formulations was evaluated using a virus neutralization test. Following the third vaccination, daily egg collection commenced. The collected eggs were sorted and labeled according to their respective groups and stored at a temperature range of 16–20 °C for further analysis.

### 2.3. Isolation of IgY from Egg Yolk

Preparation of Egg Yolks: Eggs from groups selected based on serum virus neutralization test results were used for Immunoglobulin Y (IgY) extraction. The eggs concurrent with blood sampling were pooled before processing. After cracking, the egg whites were separated, and the yolks were reserved for extraction.

IgY Extraction Procedure by In-House Method: The yolks were diluted in a 3 mM Hydrochloric Acid (HCl) solution at a 1:10 ratio. The mixture was first centrifuged at 300× *g* rpm (Megafuge 16R; ThermoFisher Scientific, Waltham, MA, USA) for 30 min at room temperature, providing optimal conditions for phase separation. Following this, the pH of the mixture was adjusted to 5.0 (range: 4.9–5.1) using 10% acetic acid, a critical step for protein stability. Secondary centrifugation was performed at the same speed for 30 min, followed by high-speed centrifugation at 10,000× *g* for 15 min at 4 °C. Then, (NH_4_)_2_SO_4_ was added to the supernatant at 35 *w*/*w*% and, subsequently, 65 *w*/*w*%, with centrifugation at 10,000× *g* for 15 min at 4 °C after each addition. The precipitate containing IgY was separated, resuspended in Phosphate-Buffered Saline (PBS) until the original yolk volume was restored, and stored below −15°C.

IgY Extraction Procedure by Commercial Kit: Egg yolks were processed for IgY extraction using the Pierce™ Chicken IgY Purification Kit (Thermo Fisher Scientific, Waltham, MA, USA, Cat. No. 89835), in accordance with the manufacturer’s instructions. The samples were stored at −20 °C until use.

### 2.4. Analysis of Egg Yolk Extracts

The assessment of the total protein content in the IgY extracts was performed utilizing the Bicinchoninic Acid (BCA) assay. Furthermore, the purity of these extracts was verified through polyacrylamide gel electrophoresis (SDS-PAGE), ensuring a thorough evaluation of the IgY antibodies in terms of both concentration and integrity.

#### 2.4.1. BCA Assay

Protein concentrations in egg yolk extracts enriched with IgY were quantified using the Bicinchoninic Acid Protein Assay Kit (Sigma-Aldrich, St. Louis, MO, USA, Cat. Nos. BCA1 and B9643), with modifications for specific assay volumes. The BCA Working Reagent was prepared by mixing Reagent A and B at a 1:8 ratio, tailored for 40, 80, and 96 wells. Bovine Serum Albumin (BSA) standards, ranging from 31.25 to 2000 µg/mL, established a standard curve. IgY samples were diluted in PBS at ratios of 10 to 500.

Assays were conducted in 96-well plates, incorporating negative (PBS) and positive (BSA standard) controls, where 25 µL of each sample or BSA standard was mixed with 200 µL of the BCA Working Reagent and incubated at 37 °C for 30 min in darkness.

Absorbance was measured at 562 nm using an iEMS Reader MF (Labsystems, Helsinki, Finland), with data analyzed by Ascent Software Version 2.6 (Labsystems, Helsinki, Finland), to calculate sample concentrations and assess extraction efficiency through statistical analyses using Python’s Pandas and NumPy libraries.

#### 2.4.2. SDS-PAGE

SDS-PAGE and Coomassie Blue staining were employed to analyze egg yolk extracts. Gels were prepared using the TGX™ FastCast™ Acrylamide Kit 7.5% (Bio-Rad, Hercules, CA, USA, Cat. No. 1610171) and cast on 1.5 mm glass plates. The sample buffer was composed of 2.5 mL 1M Tris-HCl, 1g SDS, 0.8 mL 0.1% Bromophenol Blue, 4 mL glycerol, 2 mL β-mercaptoethanol, and 0.5 mL water, adjusted to 10 mL. Running buffer (10X) included 30.3 g Tris Base, 144 g Glycine, and 10 g SDS in 1 L of water.

Protein extracts were mixed 1:1 with sample buffer, denatured at 95 °C for 5 min, and 20 µL of each sample and IgY (1 mg/mL) and Ovalbumin (OVA, 1 mg/mL) as control proteins in each gel, serving as references for band identification and comparison were loaded into the gels alongside 4 µL of Precision Plus Protein™ Standards (Bio-Rad, Hercules, CA, USA, Cat. No. 161–0374) for molecular weight marking. Electrophoresis was performed at 200 volts and 150 milliamperes for 30 min. For staining, a solution containing 0.25 g Coomassie Brilliant Blue R-250, 100 mL methanol, and 25 mL acetic acid in 125 mL was used, followed by destaining in a solution of 380 mL water, 80 mL methanol, and 40 mL acetic acid. Gels were documented using the ChemiDoc™ Imaging System (Bio-Rad, Hercules, CA, USA, Cat. No. 12003153) and analyzed with Image Lab™ Software version 2.4.0.03.

### 2.5. SARS-CoV-2 Neutralization Assay

VeroE6 cells (ATCC^®^, CRL-1586™) sourced from African green monkey kidney tissue were cultured in Dulbecco’s Modified Eagle’s Medium (Merck Cat. No. D6429) supplemented with 10% heat-inactivated fetal bovine serum (Gibco, Waltham, MA, USA, Cat. No. 16140071) and 1% Penicillin–Streptomycin (Merck Cat. No. 4333) and maintained at 37 °C with 5% CO_2_. Cells were infected with the Wuhan SARS-CoV-2 isolate (hCoV-19/Hungary/SRC_isolate_2/2020, Accession ID: EPI_ISL_483637). Before utilization in the assays, its infectious titer was quantified by a TCID50 assay. Experiments involving the active virus were performed under Biosafety Level 4 (BSL-4) conditions.

Hen sera and IgY fractions underwent two-fold serial dilution and subsequent heat inactivation at 56 °C for 30 min. The treated samples were mixed with DMEM containing 400 TCID50 of SARS-CoV-2 and incubated for 1 h at 37 °C in 96-well plates (TPP Cat. No. 92096). The assay included a positive control with 400 TCID50 of SARS-CoV-2 and a negative control consisting solely of DMEM, devoid of sera, IgY fraction, or virus.

Post-neutralization, 100 μL of the virus–serum mixture was used to infect confluent (100%) VeroE6 cells for 30 min. Subsequently, cells were maintained in 200 μL of “post-infection” culture media (DMEM with 2% FBS and 1% Pen/Strep) for three days under the same incubation conditions. The neutralizing capacity of the serum was assessed by determining the highest dilution that prevented viral infection in 50% of the wells.

### 2.6. A Syrian Golden Hamster Model for Assessing and IgY Treatment Efficacy

After careful selection of the IgY extract with the most effective neutralization capacity, in vivo experiments were initiated in Syrian golden hamsters. First, the animals were acclimatized in a BSL-4 laboratory environment where they were housed in separate cages so that neither direct nor indirect contact (such as shared airspace, food, and drinking water) was allowed. Animals were divided into three groups for the experiment, which lasted for 7 days after infection. The negative control group received PBS and DMEM. The IgY treatment group was administered IgY extract and subsequently exposed to the active virus (challenge). The positive control group was given PBS and active virus, like the IgY treatment group. Since SARS-CoV-2 is not fatal to hamsters, the experiments concluded with the euthanasia of the animals. The experiments with Syrian hamsters were conducted under an animal ethics license (License number: BA02/2000–26/2021).

#### 2.6.1. Housing and Preparation of Syrian Golden Hamsters

Specific Pathogen-Free (SPF) male Syrian golden hamsters, 5–6 weeks old, were obtained from Janvier Labs, France. Individual housing in ventilated cages (Allentown, Animal Transfer Unit) was provided, including cellulose bedding, nesting materials, and chewable wood. Animals had continuous access to food and 400 mL of tap water, and these were replenished every two days. Animals were acclimatized in a BSL-4 laboratory environment where they were housed in separate cages so that neither direct nor indirect contact (such as shared airspace, food, and drinking water) could occur. After the 4-day acclimatization period in the BSL-4 laboratory, the experiments began.

#### 2.6.2. BSL-4 Laboratory Environment and Animal Management

The BSL-4 laboratory maintained a constant temperature (23 °C) and humidity (20–40%). Isoflurane (Aeranne, Baxter Hungary, Budapest, Hungary) anesthesia was administered via a SomnoSuite (Kent Scientific, Torrington, CT, USA) anesthesia machine (induction and maintenance at 5%). An Ohaus Scout scale was employed; throat swabs made with Copan FloqSwab sticks (Cat. No. 520CS01) were used for sampling; and for euthanasia, we used retroorbital venipuncture with glass capillaries (Harvard Apparatus, Holliston, MA, USA, Cat. No. 30–0037).

#### 2.6.3. Pre-Infection Treatment and Inoculation

Hamsters were treated with IgY extract or PBS (for control groups) intranasally (10–10 μL/nostril by an automated pipette) one hour before inoculation with 180 PFU of the Wuhan strain of SARS-CoV-2 per animal. The negative control group was treated with DMEM as a mock inoculation. Virus dilution was previously prepared in DMEM (Merck, Darmstadt, Germany, Cat. No. D6429).

#### 2.6.4. Daily Observation and Post-Infection Procedure

**Figure 1:**
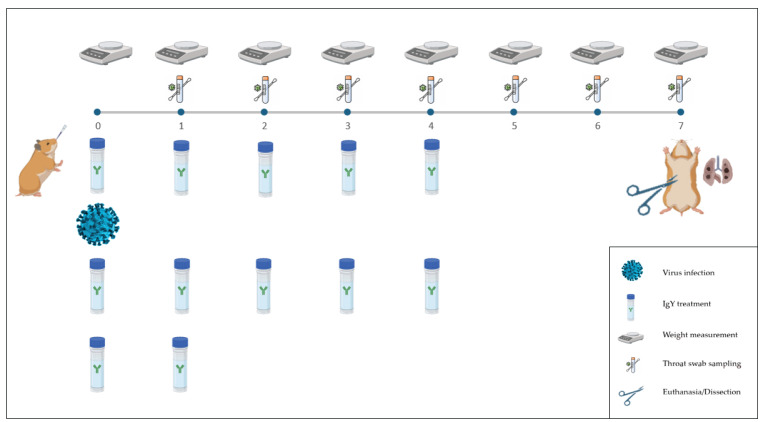
Schematic representation of the animal experimental arrangement. Syrian hamsters were treated with IgY or PBS and challenged with SARS-CoV-2. The experimental timeline is shown, where animals were weighed daily, and throat swabs were collected on days 1, 2, 3, 4, 5, and 6 post-infection. Animals were treated with IgY or PBS every 8 h for the first 3 days and every 12 h until day 4 post-infection. On day 7, animals were euthanized, and lung tissue samples were collected for further histopathological analysis. Each row depicts the timeline and the corresponding actions performed, including sample collection, treatment, and final tissue dissection.

Daily assessments were performed, which involved weight measurement, throat swab sampling, and an assessment of the animal’s general health status. IgY or PBS treatment was administered every 8 h for the first three days and then every 12 h until 4 dpi. Euthanasia was performed on day 7 post-infection via retroorbital bleeding under exsanguination (Figure 1).

### 2.7. ddPCR Analysis for SARS-CoV-2 Viral Load Quantification

Nucleic Acid Extraction: Nucleic acid extraction was performed from both throat swabs and lung tissue samples. For the throat swab samples, the medium in which the swab was initially placed was used for extraction. In the case of lung tissue samples, the samples (~50 µg) were homogenized prior to extraction. This critical step was performed to ensure efficient RNA was recovered from the tissue samples. The throat swab samples were extracted by a Zybio Nucleic Acid Extraction Kit (Zybio, Catalog No. CoV2–32, Dadukou, Chongqing, China) and for the lung tissues, the Monarch^®^ Total RNA Miniprep Kit (New England Biolabs, Ipswitch, MA, USA) was used according to the manufacturer’s instructions.

### 2.8. ddPCR Methodology for Viral Load Determination

Preparation of ddPCR Master Mix: The ddPCR master mix was composed of 1X supermix, 20 U/µL reverse transcriptase, 15 mM dithiothreitol, 11.1 µL nuclease-free water, 900 nM each of forward and reverse primers, 250 nM TaqMan probe, and 2 µL of the 100-fold diluted RNA extract. The primers and probe targeted the SARS-CoV-2 Charité/Berlin RdRp gene (Integrated DNA Technologies, Coralville, IA, USA).

Droplet Generation and PCR Amplification: Droplet generation was conducted using the QX200 Droplet Generator (Bio-Rad, Hercules, CA, USA) with a specified reaction mix volume (volume details needed). The thermal cycling in a C1000 Touch Thermal Cycler included the following steps:

- Reverse transcription at 50 °C for 60 min.
- Enzyme activation at 95 °C for 10 min.
- Forty cycles of denaturation at 95 °C for 30 s and annealing/extension at 58 °C for 1 min.
- Final enzyme deactivation at 98 °C for 10 min.
- Storage of amplicons at 4 °C until droplet reading.

Droplet Reading and Data Analysis: The amplicons were analyzed using the QX200 Droplet Reader (Bio-Rad, Hercules, CA, USA). Viral copy numbers per microliter were calculated automatically by Quantasoft™ Analysis Pro version 1.0 (Bio-Rad, Hercules, CA, USA).

### 2.9. Histopathological Analysis of Lung Tissue

Tissue Fixation and Paraffin Embedding: Lung tissues from 36 hamsters were initially fixed in a 6% neutral buffered formaldehyde solution (Molar Chemicals, Cat. No. 42322-006-340) for at least 24 h at room temperature. The fixed tissue samples were trimmed and dehydrated with ethanol and xylene in an automatic tissue processor. The dehydrated samples were embedded in paraffin blocks, and 4 µm thin sections were cut manually and mounted onto Superfrost + adhesion slides (Thermo Fisher Scientific, Waltham, MA, USA). The unstained sections were deparaffinized and rehydrated in xylene and alcohol, respectively. Routine Hematoxylin and Eosin (H and E) staining was performed in an automatic staining instrument. The slides were scanned with a Pannoramic Midi slide scanner using a 20× objective (3D Histech, Budapest, Hungary) and visualized by SlideViewer software Version 2.8 (3Dhistech, Budapest, Hungary). Representative pictures were obtained with the latter software.

#### Digital Image Analysis

To quantify the consolidated regions of the lungs, a simplified approach based on the digital workflow of Mulka et al. was implemented using QuPath digital image analysis software [19,20]. For the QuPath analysis, the digital images were further converted to WS DICOM (Digital Imaging and Communications in Medicine) format by the SlideMaster software Version 2.7.0 (3D Histech) and analyzed with QuPath (version 0.4.4) software (qupath.github.io), [19].

Tissues were annotated with the wand tool, then the DoG superpixel segmentation command was applied, and intensity features were added. Distinct annotations were created within the tissues in multiple slides to educate the classifier. These annotations were marked as “consolidated”, “normal”, “blood”, or “ignore”. The normal tissue was indicative of lung parenchyma without any lesions, and the ignore class included artifacts, atelectasis as well as the walls of large blood vessels and airways. The performance of the classifier was evaluated after each analysis. The consolidation ratio was calculated by dividing the number of consolidated superpixels by the total number of lung superpixels in each case.

### 2.10. Statistical Analysis of Data

Statistical analysis was performed on non-transformed data using jamovi (Version 2.4) and R (Version 4.1) [21,22]. The R packages used for the statistical analyses were retrieved from the Comprehensive R Archive Network (CRAN) snapshot on 2023-04-07 (YYYY-MM-DD). To compare the differences (ddPCR results, body weight, and histopathological data) between the negative control, IgY-treated, and positive control groups during the 7-day-long experiment, we used the non-parametric Kruskal–Wallis test with post hoc Dwass–Steel–Critchlow–Fligner pairwise comparisons, with a predetermined alpha level of 0.05 for statistical significance. Non-parametric tests were initially selected, since we did not expect the data to follow normal distribution in the treatment group, which was apparent after data collection.

## 3. Results

### 3.1. SARS-CoV-2 Neutralization Assay on Immunized Hens’ Sera

The sera of the hens were collected at different time points after immunization. The virus neutralization capacity of hen sera was measured by using the Wuhan isolate of SARS-CoV-2. The in vitro virus neutralization titers were used as primary endpoints of the experiments, based on which the most effective immunogen constructs were selected for the consecutive experimental steps (Figure S1).

The majority of the 18 immunogen preparations were excluded after the first round of evaluation. Certain immunogen preparations induced no or very low virus neutralization titers in immunized hens. This was particularly prevalent in the case of the sera collected from hens immunized with a preparation where the protein component was the S1 subunit. Similar results were obtained with all preparations in which the RBD was used at 1 µg quantity per animal per dose. Some immunogen preparations induced a good antibody response, especially when applied at higher (1 µg vs. 10 µg) antigen content.

**Figure 2:**
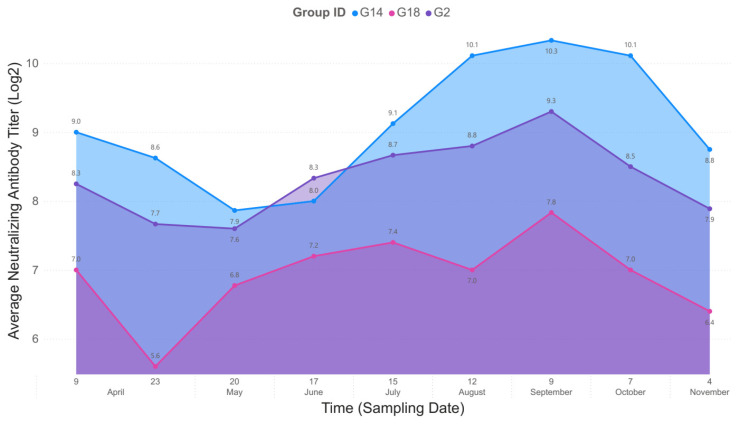
Temporal progression of average neutralizing antibody titers (Log2) across three groups: G14, G18, and G2. The y-axis represents the average neutralizing antibody titer in Log2 scale, while the x-axis displays the sampling dates over time, from April to November. Group G14 (blue) consistently exhibited the highest neutralizing titers, peaking at 10.3 Log2 in early September. Group G2 (purple) followed a similar trend, with a slightly lower peak of 9.3 Log2. Group G18 (pink) maintained the lowest titers throughout the study, peaking at 7.8 Log2 in September. The data highlight the temporal changes in neutralizing titers among the three groups, with G14 showing the strongest and most sustained response.

In the end, the eggs of three groups of hens receiving the antigen–adjuvant combinations that induced the highest virus neutralization titers were selected for egg yolk IgY examination. Hens in the G2 group that were immunized with 10 µg S protein mixed with TiterMax had a mean 8.8 log2 serum virus neutralization titer at week 2 after the second immunization and similar value (8.7 log2 titer) at week 19 after receiving the fourth immunizing dose. The groups of hens immunized with 10 µg S protein with Montanide + CpG (G14) had mean serum virus neutralization titers ranging from 9.2 log2 in the first measurement and 9.2 log2 in the last test point. Finally, the group that included hens immunized with 10 µg RBD and adjuvated with Montanide + CpG (G18) had a mean 6.4 log2 titer that changed to 6.8 log2 at the end of the test period. In all three groups, serum virus neutralization titers plateaued between the 3rd and 11th week after the fourth immunization (log2 titers: G2, 10.1; G14, 11.1; G18, 8.7, Figure 2).

### 3.2. Quantification of IgY from Egg Yolk

The eggs of the hens in the selected groups were collected, marked, and stored for further processing. The extraction of the IgY content from the eggs was performed as described above.

The virus-neutralizing activity of egg yolk origin IgY of the three selected groups of laying hens with the highest serum virus neutralization titers was tested in a similar way as described above for the testing of serum virus neutralization titers. The first egg yolk sampling was performed at week 2 after the third immunization of hens and repeated as many times as the sampling for serum antibody neutralization test was performed. The virus neutralization titer peaked at week 10 after the third dose and maintained constant values after the fourth dose was administered. At week 19 after the fourth dose, the titers decreased for G14 and G18 (log2 titers, 7) and remained constant with the G2 immunogen (log2 titer, 10). However, the highest virus neutralization titers were achieved with egg yolk IgY extracts of G14; therefore, further experiments were carried out using this product.

### 3.3. Analysis of Egg Yolk Extracts

IgY Extraction and Purity: This study evaluated the efficacy of two methods used to extract IgY from egg yolk, focusing on their yield, purity, and concentration. In group G14, the egg yolk extract exhibited a total protein content of 16.5 mg/mL, with an IgY concentration of 6.6 mg/mL and a purity rate of 40%. Despite the relevance of purity for certain applications, the intended local treatment use of these extracts deemed this factor less critical (Table S1).

Comparative Analysis of Extraction Methods: The proprietary extraction method yielded egg yolk extracts with approximately 40% purity, as opposed to the higher purity levels achieved using the commercial Pierce™ Chicken IgY Purification Kit (Thermo Fisher Scientific, Waltham, MA, USA, Cat. No: 44918). However, the in-house method consistently produced higher IgY concentrations, albeit with an increase in other protein levels. This suggests a trade-off between purity and IgY yield, with the in-house method favoring the latter (Table S1 and Figure S2).

Variability in Extraction Efficiency: A comparative study of the Pierce Kit and the in-house method revealed distinct differences in performance. The Pierce Kit maintained high purity levels (average: 87.64%, SD: 4.42%), showcasing its reliability and efficiency. Conversely, the in-house method exhibited lower purity (average: 41.88%) with greater variability (SD: 5.33%), indicating a less consistent performance (Table S8).

IgY Yield Analysis: The analysis of IgY yield, in relation to the total protein extracted, highlighted the efficiency of the methods used. Yields ranged from 82% to 96%, illustrating the high capability of the employed methods, especially the Pierce Kit, to selectively purify IgY antibodies from egg yolks (Table S8).

### 3.4. A Syrian Golden Hamster Model for Assessing IgY Treatment Efficacy

Hamsters were acclimatized during 4–6 days outside the BSL-4 facility, followed by 1–4 days inside the facility. Monitoring of specific physiological parameters began on Day 0 of the experiments.

In contrast, the weight of animals is a widely accepted parameter for infection evaluation. Generally, negative control animals are expected to gain weight, while infected animals typically experience weight loss. According to the literature, expected weight changes fall within a ± 10% range of the original weights. In our study, the overall average weight by the end of the experiment (Day 7) was 86.46 g ± 7.05 g.

**Figure 3:**
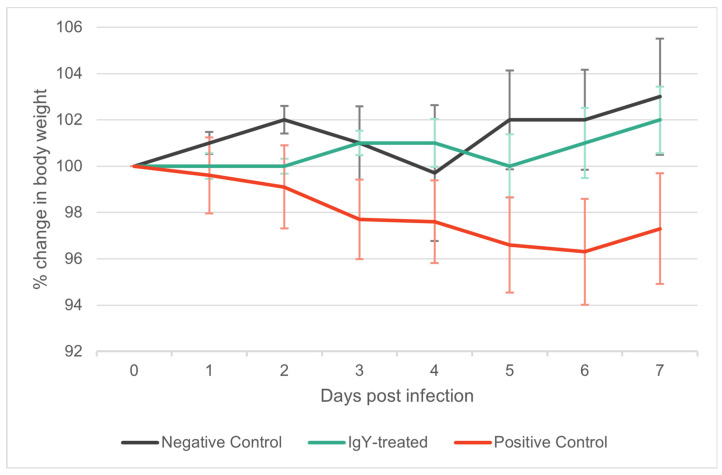
Trajectory of mean body weight changes as a percentage of Day 0 across seven days in Syrian golden hamsters. This graph illustrates the daily percentage changes in mean body weight for three groups: negative control (black line), IgY-treated (green line), and positive control (red line). Error bars indicate standard deviation, underscoring the fluctuation in weight within each group. Values over 100% indicate an overall increase in weight relative to Day 0, while values under 100% reflect a decrease, suggesting weight gain or loss trends among the experimental cohorts throughout the study period.

The IgY-treated group tended to gain weight (Day 7 mean: 101.93% of mean weight at Day 0), similarly to the negative control group (Day 7 mean: 103.16% of mean weight at Day 0). Conversely, the positive control group predominantly exhibited weight loss (Day 7 mean: 97.28% of mean weight at Day 0) (Table S2, Figure 3). The discernible trend in weight changes was exhibited by the groups, although notably pronounced weight trend differences are only anticipated with higher levels of infectious doses. For this experimental framework and considering the extent and methods through which we assess the impact of SARS-CoV-2 in the animal model (specifically through ddPCR and subsequent histopathological evaluation) the administration of a higher SARS-CoV-2 dose would yield no scientific advantage. Therefore, due to ethical considerations, we opted for a lower but still viable PFU for the experiment.

### 3.5. ddPCR Analysis for SARS-CoV-2 Viral Load Quantification

**Figure 4:**
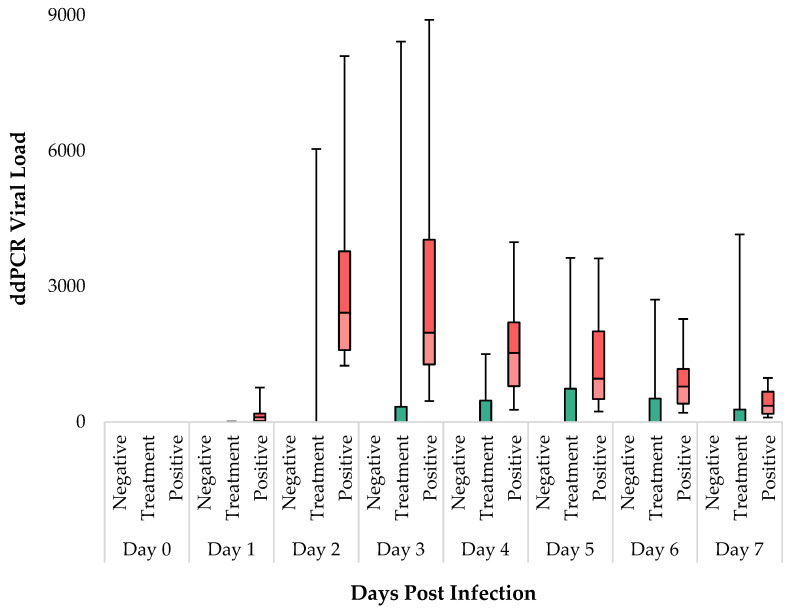
Daily ddPCR viral load analysis in Syrian golden hamsters. The boxplots represent the distribution of viral loads in three experimental groups: negative (black), treatment (green), and positive (red). Minimum and maximum values are indicated by the whiskers, the interquartile range (IQR) by the boxes, and the median by the horizontal line within each box.

The results obtained from ddPCR measurements show significant differences among the groups from Day 1. On all days of the experiment, the negative control group (*n* = 12) consistently tested negative. In the IgY-treated group (*n* = 12), 66% (eight animals) remained negative throughout, while four animals displayed signs of SARS-CoV-2 in their samples (Table S3). Conversely, in the positive control group (*n* = 12), all animals tested positive for SARS-CoV-2 from Day 1 onwards. Our data indicate that virus infection and SARS-CoV-2 levels peaked on Days 2 and 3. Overall, the IgY-treated group exhibited a noticeably lower average copy number compared to the positive control (Table S4). The Kruskal–Wallis test was employed, and significant differences were observed between the groups from Day 1 to Day 7 (Table S5). DSCF pairwise comparison post hoc analysis showed no significant differences between the negative control and IgY-treated groups (α = 0.05). However, the ddPCR copy numbers in the positive control group were consistently significantly higher compared to both the negative control, and the IgY-treated group throughout the experiment. Notably, from Day 1 to Day 5, the difference in copy number between the positive control group and IgY-treated groups was significant, while on Days 6 and 7, it was not (Table S6 and Figure 4). The positive control group thus exhibited a mean value with a standard deviation (SD) that differed from that of the IgY-treated group. However, as detailed before within the IgY-treated group (*n* = 12), eight animals displayed essentially zero copy numbers during the 7 days, while four had comparable copy numbers to those of the positive control group. These data distribution within the IgY-treated group introduces a certain level of statistical bias. Nonetheless, while acknowledging this limitation, we deemed conducting a subgroup analysis within the IgY-treated group unreasonable, since there was a very low number of animals in this subgroup.

### 3.6. Lung Tissue Histopathological Analysis

**Figure 5:**
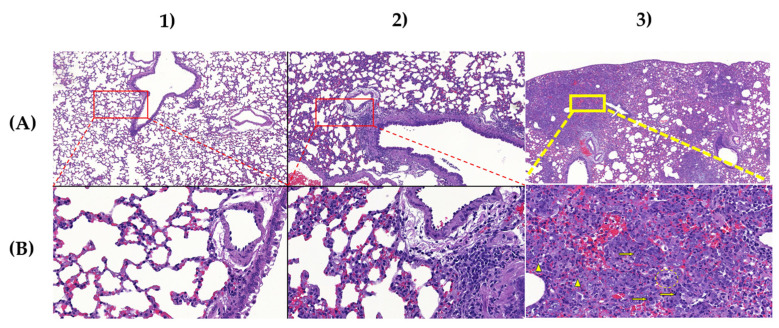
Representative low- (**A**) and high-power (**B**) pictures from negative control (1), IgY-treated, (2) and positive control (3) animals. No microscopical changes can be seen in the lung tissue of the negative control animal, whereas only minimal to mild perivascular and peribronchial mononuclear cell infiltration and minimal alveolar wall thickening can be observed in the IgY-treated animal. The positive control animal shows multifocal, coalescing areas of severe interstitial pneumonia causing visible consolidation. Numerous mitotic figures were observed among the hyperplastic type II pneumocytes (arrows), whereas degenerating neutrophil granulocytes (arrowheads) and foamy macrophages (dashed circle) were seen in the alveoli. Original magnification: (**A**): 10×; (**B**): 70×.

Animals in the positive control group showed various degree of lung consolidation consisting of type II pneumocyte hyperplasia with the presence of numerous mitotic figures, interstitial and intra-alveolar accumulation of macrophages, lymphocytes, and a few neutrophil granulocytes indicating interstitial pneumonia. Lymphocytic perivasculitis with perivascular edema was also observed. The IgY-treated animals showed similar, but substantially less severe lesions compared to the non-treated group. Animals in the negative control group showed only background or procedure-related changes, such as congestion and alveolar emphysema (Figure 5).

**Figure 6:**
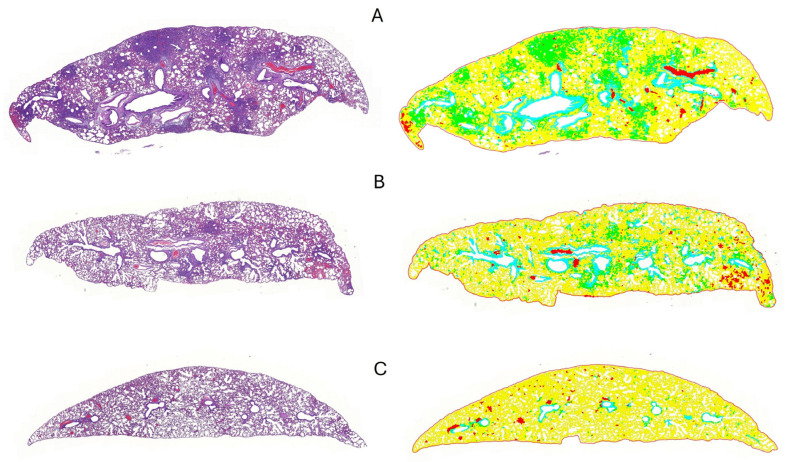
Representative images of lung tissues from Syrian hamsters in different treatment groups. The left column shows Hematoxylin and Eosin (H and E)-stained lung sections at low magnification (2.8×), while the right column displays QuPath-generated color-coded areas. In the color-coded images, green areas indicate lung tissue consolidation and yellow indicates normal lung parenchyma. (**A**) The positive control group shows severe, multifocal, coalescing areas of consolidation; (**B**) the IgY-treated group displays mild, multifocal areas of consolidation; (**C**) the negative control group exhibits no significant consolidation, reflecting normal lung tissue.

**Figure 7:**
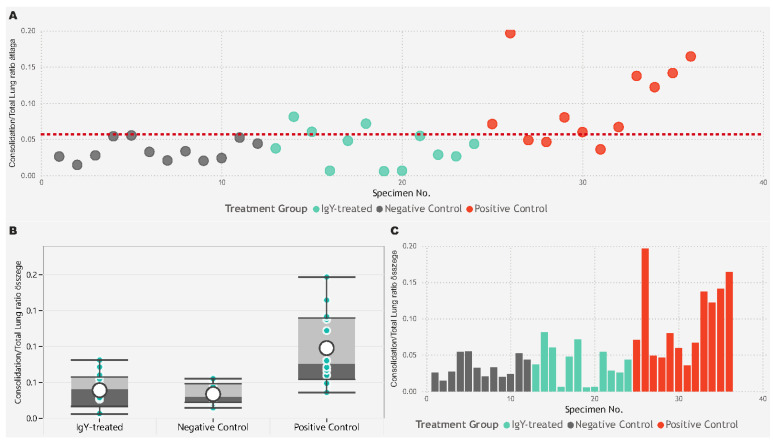
The image displays three plots analyzing the consolidation to total lung area ratio across different treatment groups. (**A**) The scatter plot shows the consolidation/total lung ratio for individual specimens, with each point color-coded by treatment group (IgY-treated in green *n* = 12, negative control in gray *n* = 12, and positive control in red *n* = 12). A horizontal dashed red line represents the reference value for the average consolidation/total lung ratio. The x-axis denotes the specimen number, while the y-axis indicates the consolidation ratio. (**B**) The boxplot provides a summary of the consolidation/total lung ratios for each treatment group, with overlaid individual data points. The box illustrates the median, interquartile range, and potential outliers. (**C**) The bar plot shows the sum of the consolidation/total lung ratios for each specimen, grouped by treatment group. The data indicate that the positive control group has higher consolidation/total lung ratios compared to the other groups.

We used digital image analysis to objectively quantify the percentage of lung areas affected by consolidation consisting of interstitial pneumonia and type II pneumocyte hyperplasia in the infected animals (Figure 6). The Kruskal–Wallis test was used to identify differences in the percentage of consolidation to total lung area pixel density data across all groups. The test indicated that at least one group differed significantly from the others (*p* < 0.001, ε^2^ = 0.4032). Following this, we conducted Dwass–Steel–Critchlow–Fligner pairwise comparisons to pinpoint the specific group differences. This analysis revealed that the data from the IgY-treated group did not significantly differ from the negative control group (*p* = 0.801). However, both the IgY-treated group and the negative control group were significantly different from the positive control group (*p* = 0.001 and *p* = 0.0015, respectively) (Figure 7).

Note: Effect sizes were categorized as small (ε^2^ < 0.01), medium (0.01 ≤ ε^2^ < 0.06), and large (ε^2^ ≥ 0.06), which corresponded to minimal, moderate, and substantial practical significance, respectively. Detailed individual scores and pairwise comparison results are available in the Supplementary Tables (Table S7).

## 4. Discussion

The initially uncontrollable spread and massive disease burden of COVID-19 led to concerted research and development efforts worldwide. This unprecedented global health emergency demanded innovative approaches in drug and vaccine development strategies [23,24]. The realization that eliciting an immune response by vaccination is unlikely to always be effective for or available to everyone sparked interest in the development of products which provide safe and effective passive immunity. The attention of researchers was partly focused on IgY-based therapy [16,25]. IgY antibodies, derived from egg yolks, offer several advantages over traditional IgG-based therapies; these include cost-effective production, enhanced stability, and the reduced risk of adverse immune responses, making them a promising alternative for combating viral infections. Furthermore, IgY is far more suitable for industrial-scale production than most other therapeutic antibodies [18,26,27].

In recent years, several research papers have reported the potential efficacy of IgY against SARS-CoV-2 infection [28,29,30,31]. For example, published data indicated that egg yolk-derived IgY recognizes and cross-neutralizes diverse SARS-CoV-2 variants and nasal administration of IgY products may have an overall positive effect on the outcome of acute infection [28,29,32,33]. The present study reinforces earlier observations concerning the effectiveness of IgY antibodies in the reduction of viral load and improvement of respiratory pathology by using a Syrian hamster model. These results suggest the potential of egg yolk-derived IgY as a pre- and post-exposure therapeutic tool against COVID-19, especially for individuals who are immunocompromised or cannot access vaccines.

As we noticed in the literature, in previous SARS-CoV-2-based IgY therapy studies, researchers wrote succinctly about the optimization of the antigen–adjuvant formulations used in hen immunization [29,34,35]. As there is increasing interest in the utilization of egg(-derived) products in biomedicine, the authors felt it important to conduct experiments to maximize the IgY response and improve the product quality for future formulation design. The results of the virus neutralization assay showed that hens immunized with higher doses of spike protein or receptor-binding domain (RBD) antigens, particularly when combined with potent adjuvants like Montanide and CpG oligonucleotide, produced strong neutralizing antibody responses. Notably, the G14 group, which received 10 µg of full-length spike protein with Montanide + CpG adjuvant, exhibited the highest virus neutralization titers. Nonetheless, experimental data concerning the superiority of full-length S protein as an antigen over other S protein domains (e.g., RBD or S2 protein) were not evident in other studies [32,36]. Therefore, further investigation on the optimal combination of antigen type, antigen dose, and adjuvant formulation is still worthwhile, as it may be possible to achieve a more robust immune response in hens.

The industry-scale production of therapeutic IgY products requires the use of cost-effective procedures that avoid the use of toxic substances. Morgan et al. (2021) [37] provided an overview of the most widely used IgY extraction techniques, including commercial solutions. Some of the readily scalable methods (e.g., water dilution and PEG precipitation) are commonly used in relevant SARS-CoV-2 studies. In our study, we did not aim to compare all available methods, mainly because each of them would have required separate laboratory optimization. Instead, we used and compared a commercial IgY extraction method and an in-house IgY precipitation method. The comparison of these two IgY extraction methods showed that the in-house technique produced higher amounts of IgY but lower purity than the commercial purification kit. However, since the antibodies are intended for topical or mucosal use, the level of purity was less critical. We believe that our modified extraction technique (patent application identifier, P 22 00361), which allows for the efficient production of SARS-CoV-2-specific IgY antibodies with potent neutralizing activity, represents a significant advancement in future immunotherapy and viral infection management.

Unlike monoclonal antibodies or traditional immunotherapies (e.g., anti-COVID-19 horse serum), IgY antibodies offer several unique advantages, particularly their large-scale production from egg yolks, making them highly scalable and cost-effective for high-dose applications. Additionally, IgY antibodies lack the Fc region present in mammalian antibodies, reducing the risk of Fc receptor-mediated inflammatory responses, which can be a concern with some IgG-based therapies. In contrast, equine-based serotherapies have demonstrated potent neutralizing effects but come with higher risks of hypersensitivity reactions and are often less suitable for repeated administration due to immunogenicity concerns [38].

Rodent models used in IgY therapy projects against SARS-CoV-2 showed variable in vivo efficacy along different parameters [29,36,39,40]. In this study, we used Syrian golden hamster in “in vivo” experiments. In this animal model, ddPCR analysis for virus detection confirmed that IgY treatment significantly reduced SARS-CoV-2 viral loads compared to the positive control group (in which the animals received no IgY treatment). The IgY-treated animals showed a markedly lower viral load, with 66% of the animals in the treatment group remaining virus-free throughout the experiment. This finding led us to conclude that IgY could effectively neutralize the virus at the cellular entry point, thereby preventing severe infection, a finding that Wei and coworkers already hypothesized in their study [41]. In addition, the lung histopathological analysis showed that IgY-treated animals experienced substantially less tissue consolidation and inflammation than untreated animals, further supporting the protective role of IgY in mitigating the impact of SARS-CoV-2, which was previously described by Zhao et al. [27]. The digital image ana-lysis of lung tissues provided a quantifiable measure of the treatment efficacy, showing significant differences between the IgY-treated and positive control groups in terms of lung consolidation ratios. The reduced lung damage in the IgY-treated group demonstrates the therapeutic potential of IgY in protecting against COVID-19-associated pneumonia, one of the most severe complications of the disease.

## 5. Conclusions

Our investigation into IgY-mediated prophylaxis against SARS-CoV-2 reveals the potential and feasibility of a safe, scalable, and ethically sourced prophylactic strategy that can serve as a complement to, or an alternative to, traditional therapeutic approaches. This study demonstrates that IgY antibodies, specifically those targeting the SARS-CoV-2 spike protein, can effectively inhibit viral replication and alleviate disease pathology in a relevant animal model. Further optimization of the specific dosage, frequency, and duration of IgY application could help address the therapeutic needs of individuals for whom vaccination is not feasible or effective. These promising results establish a strong foundation for future research exploring the role of IgY antibodies in combating not only COVID-19 but also a broader spectrum of respiratory viral infections.

## 6. Patents

The work reported in this manuscript has led to the filing of a patent application, which is disclosed in the “*Szabadalmi Közlöny és Védjegyértesítő*”, the official journal of the National Office of Intellectual Property of Hungary, volume 128, issue 18, dated 28 September 2023. The patent application, identified by the number P 22 00361, filed on 9 September 2022, by PROPHYL Animal Health, Diagnostics, Research, and Service Limited Liability Company, is centered around an IgY antibody with potent in vitro neutralizing activity against SARS-CoV-2 antigens, rendering it suitable for application in combating viral infections.

The inventors listed for this patent are Dr. Szabóné Dr. Benyeda Zsófia, Dr. Palya Vilmos, Dr. Nemes Csaba Miklós, Dr. Bajnóczi Pál, and Faragó-Sipos Orsolya, who have contributed to the development of this IgY antibody. Their invention encompasses the use of this antibody in the treatment of viral infections, highlighting its potential application in managing and preventing diseases caused by SARS-CoV-2.

This patent application represents a significant advancement in the field of immunotherapy and infectious disease control, providing a novel approach to addressing the ongoing challenges posed by COVID-19 and potentially other viral pathogens. The development and application of this IgY antibody could offer a new line of defense against viral infections, complementing existing vaccines and therapeutic strategies.

## Figures and Tables

**Table 1:** Combinations of antigens and adjuvants used in vaccine formulation. This table outlines the structured grouping (G1–G18) based on the type of adjuvant and antigen used, along with the antigen dosage per vaccine dose. Three antigens—spike protein (S), spike protein subunit 1 (S1), and the receptor-binding domain (RBD)—were tested in two concentrations (1 µg and 10 µg) with TiterMax Gold, Montanide, and a combination of Montanide and CpG oligonucleotide as adjuvants. A total of 18 vaccine formulations were prepared for immunization studies in chickens to assess efficacy. Antigens were procured as recombinant proteins with a HIS-tag from HEK 293 cells with catalog numbers SPNC52H3 for S, S1NC52H4 for S1, and SPDC52H5 for RBD. TiterMax Gold and MontanideTM ISA 71R VG served as the primary adjuvants, with CpG oligonucleotide enhancing the immunogenic response in selected formulations. Solvent name: Aqua destillata pro injection.

Group ID	Adjuvant	Antigen	Antigen Quantity
G1	TiterMax Gold	S	1 µg
G2	TiterMax Gold	S	10 µg
G3	TiterMax Gold	S1	1 µg
G4	TiterMax Gold	S1	10 µg
G5	TiterMax Gold	RBD	1 µg
G6	TiterMax Gold	RBD	10 µg
G7	Montanide	S	1 µg
G8	Montanide	S	10 µg
G9	Montanide	S1	1 µg
G10	Montanide	S1	10 µg
G11	Montanide	RBD	1 µg
G12	Montanide	RBD	10 µg
G13	Montanide + CpG	S	1 µg
G14	Montanide + CpG	S	10 µg
G15	Montanide + CpG	S1	1 µg
G16	Montanide + CpG	S1	10 µg
G17	Montanide + CpG	RBD	1 µg
G18	Montanide + CpG	RBD	10 µg

Note: The table outlines the structured grouping (G1–G18) based on the type of adjuvant and antigen used, along with the antigen dosage per vaccine dose. Three antigens—spike protein (S), spike protein subunit 1 (S1), and the receptor-binding domain (RBD)—were tested in two concentrations (1 µg and 10 µg) with TiterMax Gold, Montanide, and a combination of Montanide and CpG oligonucleotide as adjuvants. A total of 18 vaccine formulations were prepared for immunization studies in chickens to assess efficacy. Antigens were procured as recombinant proteins with a HIS-tag from HEK 293 cells with catalog numbers SPNC52H3 for S, S1NC52H4 for S1, and SPDC52H5 for RBD. TiterMax Gold and MontanideTM ISA 71R VG served as the primary adjuvants, with CpG oligonucleotide enhancing the immunogenic response in selected formulations. Solvent name: Aqua destillata pro injection.

## Data Availability

The raw data supporting the conclusions of this study are available on request from the corresponding authors. Due to privacy and ethical restrictions related to the handling of animal models, the data cannot be made publicly accessible. Further inquiries regarding the datasets can be directed to the corresponding authors.

## Supplementary Materials

The following supporting information can be downloaded at https://www.mdpi.com/article/10.3390/vaccines12121422/s1. Figure S1: Evaluation of virus neutralization in a 96-well plate format. Figure S2: Comparative SDS-PAGE analysis of IgY purification and protein complexes. Table S1: Comparative analysis of egg yolk extracts using different extraction methods. Table S2: Body weight changes in Syrian golden hamsters over a 7-day period post-experiment. Table S3: The virus copy numbers by ddPCR related to individual hamsters. Table S4: Descriptive statistics of ddPCR viral copy numbers over time by treatment group. Table S5: Daily viral load evaluation by ddPCR in three experimental hamster groups. Table S6: Daily pairwise comparison of viral load in hamster groups using the Dwass–Steel–Critchlow–Fligner method. Table S7: This table presents the results of a Kruskal–Wallis test and subsequent pairwise comparisons for the consolidation to total lung area ratio data. Table S8. The quantification of IgY from egg yolk data for statistical analysis.
