# Supplementary material for: Evaluating the Protective Role of Intranasally Administered Avian-Derived IgY Against SARS-CoV-2 in Syrian Hamster Models
Source: Vaccines (Basel). 2024 Dec 17;12(12):1422. doi: 10.3390/vaccines12121422 (PMC11728625; doi:10.3390/vaccines12121422)
Supplement: Supplementary file 1 [file vaccines-12-01422-s001.zip › vaccines-3276984-supplementary.pdf]

Supplementary

Figures

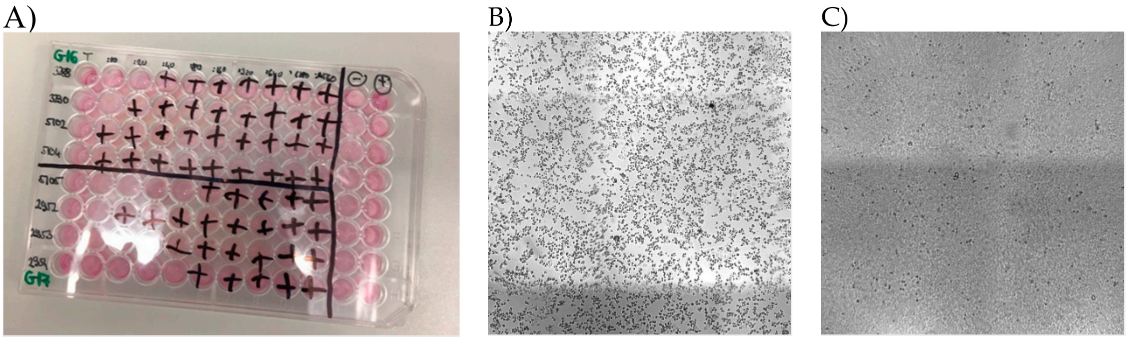

Figure S1. Evaluation of virus neutralization in a 96-well plate format. (A) 96-well plate with wells annotated for different dilutions and controls, displaying colorimetric readouts indicative of cytopathic effects. (B) Positive cytopathic effect evidenced by extensive cell lysis and viral replication, denoting a lack of neutralizing activity. (C) Negative cytopathic effect with a majority of intact cells, indicating effective viral neutralization by the tested antibodies or compounds.

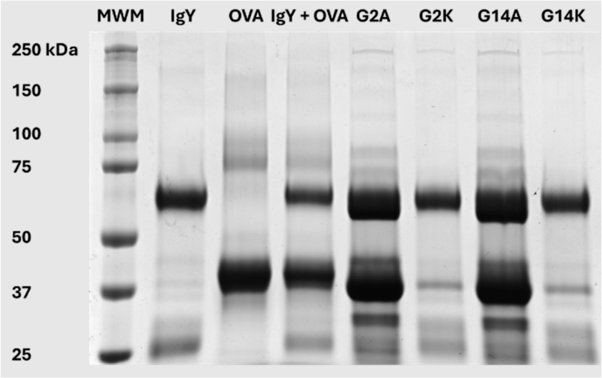

Figure S2. Comparative SDS-PAGE analysis of IgY Purification and Protein Complexes: The lanes from left to right include the molecular weight marker (MWM), pure IgY, ovalbumin (OVA), a combination of IgY and OVA, and samples from groups G2 and G14 subjected to two different purification processes, labeled as G2A (in-house method) and G2K (Pierce Chicken IgY Purification Kit) for group 2, and similarly G14A and G14K for group 14. The protein bands were stained with Coomassie Blue.

Tables

Table S1. Comparative analysis of egg yolk extracts using different extraction methods. This table summarizes the total volume, purity, total protein, and IgY concentrations of extracts obtained using the proprietary In-house Method and the IgY Purification Kit (Pierce Chicken IgY Purification Kit). The results highlight variations in IgY yield and purity between the two methods, demonstrating the efficiency of the in-house technique in generating higher IgY concentrations despite lower purity levels compared to the commercial kit.

ID	IgY Extraction Method	Total Protein (mg/ml)	IgY (mg/ml)	Purity (%)
G2/K 081213	IgY Purification Kit	6,5	5,33	82
G2/A 081213	In-House Method	18,9	10,21	54
G2/K 091214	IgY Purification Kit	4,9	4,17	85
G2/A 091214	In-House Method	14,7	6,32	43
G2/K 100911	IgY Purification Kit	5,7	4,79	84
G2/A 100911	In-House Method	21,5	8,82	41
G14/K 081213	IgY Purification Kit	7,2	6,045	84
G14/A 081213	In-House Method	25	9,75	39
G14/K 091214	IgY Purification Kit	5,1	4,39	86
G14/A 091214	In-House Method	16,5	6,60	40
G14/K 100911	IgY Purification Kit	5,2	4,37	84
G14/A 100911	In-House Method	22,2	7,99	36
G18/K 0912	IgY Purification Kit	5	4,15	83
G18/A 0912	In-House Method	15	6,30	42
G18/K 100911	IgY Purification Kit	7,6	6,38	84
G18/A 100911	In-House Method	24,9	9,96	40

Table S2. Body weight changes in Syrian golden hamsters over a 7-day period post-experiment. The table presents the mean body weights (in grams) and percentage changes from Day 0 for three groups: Negative control, IgY treated, and positive control group. Standard deviations are provided for each measurement to indicate variability within groups. Data are represented to reflect weight dynamics across the experimental timeline, highlighting the physiological impact of treatments on animal weight.

	Treatment Group	Day 0	Day 1	Day 2	Day 3	Day 4	Day 5	Day 6	Day 7
**Mean**	Negative Control	88.2 (100 %)	89.3 (101 %)	89.8 (102 %)	89.5 (101 %)	87.9 (99.7 %)	90 (102 %)	90.1 (102 %)	91 (103 %)
	IgY Treated	84 (100 %)	84.2 (100 %)	84.3 (100 %)	84.5 (101 %)	84.6 (101 %)	84.4 (100 %)	84.7 (101 %)	85.7 (102 %)
	Positive Control	85.1 (100 %)	84.6 (99.6 %)	84.3 (99.1 %)	83 (97.7 %)	82.9 (97.6 %)	82.1 (96.6 %)	81.8 (96.3 %)	82.7 (97.3 %)
**Standard Deviation**	Negative Control	4.48 (5.08 %)	4.71 (5.34 %)	4.35 (4.93 %)	4.16 (4.72 %)	5.55 (6.29 %)	4.77 (5.41 %)	4.98 (5.65 %)	6.08 (6.89 %)
	IgY Treated	5.36 (6.38 %)	5.35 (6.37 %)	5.32 (6.33 %)	5.38 (6.4 %)	5.68 (6.76 %)	5.95 (7.08 %)	6.13 (7.3 %)	5.9 (7.02 %)
	Positive Control	6.92 (8.13 %)	5.91 (6.94 %)	6.12 (7.19 %)	6.1 (7.17 %)	5.99 (7.04 %)	5.77 (6.78 %)	6.28 (7.38 %)	6.92 (8.13 %)

Specimen No.	Treatment Group	Weight Day0	Weight Day1	Weight Day2	Weight Day3	Weight Day4	Weight Day5	Weight Day6	Weight Day7
1	Negative Control	91,6	91,1	92,8	94,3	95,1	95,1	91,6	94,3
2	Negative Control	83,9	85,6	86,4	86,3	86,6	84,8	84	84,7
3	Negative Control	93,2	94,7	95,3	96,7	98,1	99,2	100,1	101,7
4	Negative Control	82,3	83,5	83,5	84,4	84,7	85,2	86,2	87,9
5	Negative Control	86,5	88,7	89,4	90,4	92,7	93,6	95,2	96,9
6	Negative Control	88,5	89,8	90,9	92,4	92,1	93,3	93,6	94,9
7	Negative Control	82,6	82,7	84,3	81,5	78	82,1	81,7	78,6
8	Negative Control	87,5	89	90,4	88,7	86,1	89,4	89,6	89,6
9	Negative Control	87	86,9	86,8	89,2	86,6	88,6	89,1	89,9
10	Negative Control	86	87	86,5	88,5	85	89,6	88,5	88,2
11	Negative Control	93,7	95,4	94,4	89,7	84	89,1	89,7	90,2
12	Negative Control	95,9	97,8	96,5	91,5	85,9	90,1	92,4	95,1
13	IgY Treated	84,5	84,2	84,5	84,4	86,4	86	86,7	86
14	IgY Treated	84,7	84,4	84,6	83,5	81,2	79,6	78,7	80,3
15	IgY Treated	92,6	94,3	93,4	93,6	94	93,9	94,8	95,2
16	IgY Treated	78,4	79,2	79,5	80	81,3	81,6	81,1	82,5
17	IgY Treated	82,6	83,4	82,4	83,2	82,2	81,8	83,8	84,4
18	IgY Treated	78,6	79,4	78,9	79,5	78,4	76,5	77,1	79
19	IgY Treated	83,9	82,2	83,1	82,9	82,8	82,6	82,3	83,1
20	IgY Treated	79,8	79,4	79,8	79,9	79,4	80,7	80,6	80,9
21	IgY Treated	78,9	79,9	79,7	80,1	80,2	80,6	81,2	82,7
22	IgY Treated	83,1	83,3	83,7	84,1	85,2	85,2	85,3	86,7
23	IgY Treated	86,1	85,4	86,3	86,1	87,4	86,8	87,7	89
24	IgY Treated	95,4	95	95,5	96,4	96,4	97	97,2	98,2
25	Positive Control	92,3	92,7	93	92	92,7	91,8	94,4	96,6
26	Positive Control	77,8	78,8	79,1	77,6	78,4	76,1	73,4	73,5
27	Positive Control	81	82,1	80,5	79,3	78,7	76,9	77,3	77,9
28	Positive Control	81,9	82,2	81,7	81,1	82,4	84,2	83,7	83,6
29	Positive Control	82,7	84,6	84	83	81,6	80,7	82,3	83,9
30	Positive Control	89,6	90,6	90,1	87,6	86,9	82,6	80,8	81,9
31	Positive Control	80,2	79,5	79,4	78,2	77,6	77,6	78,5	80,1
32	Positive Control	92,1	92,4	93,2	92,6	91,7	90,2	89,3	91,5
33	Positive Control	97,8	87,6	86,3	85,6	85,3	84,3	84,5	85,5
34	Positive Control	74,7	74,1	73,4	72,2	72,6	73,2	72,1	71,9
35	Positive Control	82	81,7	81,6	80,5	80,1	80	80,9	80,5
36	Positive Control	89,2	89,4	88,9	86,9	87,1	87,4	85	85,7

Table S3. The virus copy numbers by ddPCR related to individual hamsters.

		ddPCR	ddPCR	ddPCR	ddPCR	ddPCR	ddPCR	ddPCR	ddPCR
Specimen No.	Treatment Group	Day0	Day1	Day2	Day3	Day4	Day5	Day6	Day7
1	Negative Control	0	0	0	0	0	0	0	0
2	Negative Control	0	0	0	0	0	0	0	0
3	Negative Control	0	0	0	0	0	0	0	0
4	Negative Control	0	0	0	0	0	0	0	0
5	Negative Control	0	0	0	0	0	0	0	0
6	Negative Control	0	0	0	0	0	0	0	0
7	Negative Control	0	0	0	0	0	0	0	0
8	Negative Control	0	0	0	0	0	0	0	0
9	Negative Control	0	0	0	0	0	0	0	0
10	Negative Control	0	0	0	0	0	0	0	0
11	Negative Control	0	0	0	0	0	0	0	0
12	Negative Control	0	0	0	0	0	0	0	0
13	IgY Treated	0	0	0	0	0	0	0	0
14	IgY Treated	0	0	6040	8420	1502	3630	2529	432
15	IgY Treated	0	0	0	3,4	1461	948	2707	4150
16	IgY Treated	0	0	0	0	0	0	0	0
17	IgY Treated	0	8,2	9200	1338	305	715	586	468
18	IgY Treated	0	0	37	3630	974	796	493	224
19	IgY Treated	0	0	0	0	0	0	0	0
20	IgY Treated	0	0	0	0	0	0	0	0
21	IgY Treated	0	0	0	0	0	0	0	0
22	IgY Treated	0	0	0	0	0	0	0	0
23	IgY Treated	0	0	0	0	0	0	0	0
24	IgY Treated	0	0	0	0	0	0	0	0
25	Positive Control	0	162,1	2532	1681	306	3620	202	98,2
26	Positive Control	0	171	3910	1265	301	534	1263	112,4
27	Positive Control	0	17,3	1374	6950	1417	229	1140	972
28	Positive Control	0	73,2	3640	18830	3400	2130	879	960
29	Positive Control	0	5,9	1406	8900	268	396	778	430
30	Positive Control	0	131,1	2334	5430	949	925	291	237
31	Positive Control	0	760	2414	1020	2131	668	785	155
32	Positive Control	0	3,7	1770	2105	3980	985	544	441
33	Positive Control	0	221	20047	1972	1621	1271	2277	621
34	Positive Control	0	559	4160	461	2415	2360	1338	801
35	Positive Control	0	58,6	1244	1278	1653	418	377	281
36	Positive Control	0	16,2	8100	2634	1431	1962	409	191

Table S4. Descriptive statistics of ddPCR viral copy numbers over time by treatment group. This table presents the mean ddPCR viral copy numbers for each day of the experiment across three different treatment groups: Negative Control, IgY Treated, and Positive Control. The mean values indicate the average viral load detected in each group, while the standard deviations reflect the variability within each group. The Negative Control group consistently showed no viral presence, while the IgY Treated and Positive Control groups demonstrated varying levels of viral RNA copies, with the Positive Control group displaying significantly higher means and variability in viral loads (Table S2).

	**Treatment Group**	**Day 0**	**Day 1**	**Day 2**	**Day 3**	**Day 4**	**Day 5**	**Day 6**	**Day 7**
**Mean**	Negative Control	0	0	0	0	0	0	0	0
	IgY Treated	0	1	1273	1116	354	507	526	440
	Positive Control	0	182	4411	4377	1656	1292	857	442
**Standard deviation**	Negative Control	0	0	0	0	0	0	0	0
	IgY Treated	0	2	3040	2539	598	1050	999	1182
	Positive Control	0	238	5275	5259	1185	1027	586	323

Table S5. Daily viral load evaluation by ddPCR in three experimental hamster groups. The results from the Kruskal- Wallis test provide a comprehensive overview of viral load variances among the Negative Control, IgY Treated, and Positive Control groups over a span of seven days. The table delineates the chi-square (χ^2) values, degrees of freedom (df), p-values, and effect sizes (ε^2), with the latter quantifying the magnitude of observed differences. Statistically significant deviations are marked with asterisks (*) where the inter-group differences surpassed the threshold of $\alpha = 0.05$, signifying notable changes in viral load that could be attributed to treatment impact.

	χ^2	df	p	ε^2
**Day 0**	NaN	2	NaN	NaN
**Day 1**	30.8	2	< .001*	0.88
**Day 2**	25.9	2	< .001*	0.76
**Day 3**	22.4	2	< .001*	0.641
**Day 4**	23.5	2	< .001*	0.673
**Day 5**	20.9	2	< .001*	0.597
**Day 6**	19.7	2	< .001*	0.563
**Day 7**	19.9	2	< .001*	0.584

Table S6. Daily pairwise comparison of viral load in hamster groups using the Dwass–Steel–Critchlow–Fligner method. This table presents the pairwise comparison results between the Negative Control, IgY Treated, and Positive Control groups across eight days. Asterisks (*) indicate days where the differences in viral loads were statistically significant at the 5% level ($\alpha = 0.05$).

Group	Group	Day 0	Day 1	Day 2	Day 3	Day 4	Day 5	Day 6	Day 7
**Negative Control**	**IgY Treated**	NaN	0.577	0.286	0.083	0.083	0.083	0.083	0.083
**Negative Control**	**Positive Control**	NaN	< .001 *	< .001 *	< .001 *	< .001 *	< .001 *	< .001 *	< .001 *
**IgY Treated**	**Positive Control**	NaN	< .001 *	0.001 *	0.009 *	0.004 *	0.026 *	0.057	0.038 *

Table S7. This table presents the results of a Kruskal–Wallis test and subsequent pairwise comparisons for the consolidation to total lung area ratio data. The top section shows the Kruskal-Wallis test results, indicating a chi-square value (χ^2) of 14.11, with 2 degrees of freedom (df), and a highly significant p-value (< 0.001). The effect size (ε^2) is 0.4032, categorized as large based on the provided criteria (small effect: $\varepsilon^2 < 0.01$, medium effect: $0.01 \leq \varepsilon^2 < 0.06$, large effect: $\varepsilon^2 \geq 0.06$). The bottom section details the pairwise comparisons using the W statistic and p-values for three group pairs: Negative Control vs. IgY treated, Negative Control vs. Positive Control, and IgY treated vs. Positive Control.

**Kruskal-Wallis Test**				
	χ^2	**df**	**p**	ε^2
**T/L ratio**	14.11	2	< .001*	0.4032

**Pairwise comparisons - Consolidation/Total Lung ratio**				
			**W**	**p**
Neg	vs	IgY	0.898	0.801
Neg	vs	Pos	5.062	0.001*
IgY	vs	Pos	3.919	0.015*

*p<0.05 indicates statistical significance.

Specimen No.	Treatment Group	Consolidation/Total Lung ratio
1	Negative Control	0,026146646
2	Negative Control	0,014568008
3	Negative Control	0,027606699
4	Negative Control	0,054103833
5	Negative Control	0,055260941
6	Negative Control	0,032495471
7	Negative Control	0,02066487
8	Negative Control	0,033463138
9	Negative Control	0,020216738
10	Negative Control	0,023951752
11	Negative Control	0,052325581
12	Negative Control	0,044023736
13	IgY Treated	0,037401923
14	IgY Treated	0,081064356
15	IgY Treated	0,060245045
16	IgY Treated	0,006402672
17	IgY Treated	0,04788716
18	IgY Treated	0,071490194
19	IgY Treated	0,005744192
20	IgY Treated	0,006523408
21	IgY Treated	0,054636171
22	IgY Treated	0,028574701
23	IgY Treated	0,026344567
24	IgY Treated	0,043578978
25	Positive Control	0,071059201
26	Positive Control	0,196703707
27	Positive Control	0,048762234
28	Positive Control	0,046263345
29	Positive Control	0,08022323
30	Positive Control	0,059908758
31	Positive Control	0,035996087
32	Positive Control	0,066836149
33	Positive Control	0,137567117
34	Positive Control	0,121996463
35	Positive Control	0,141495042
36	Positive Control	0,164511705

Table S8. The quantification of IgY from egg yolk data for statistical analysis.

Group ID	ID	IgY Extraction Method	Total Volume (ml)	Purity (%)	Total Protein (mg/ml)	IgY concentration (mg/ml)	Total Protein Quantity (mg)	Total IgY Quantity (mg)
G2	G2/A 081213	HCl-2xAS method	15	54	18,9	10,21	283,5	153,15
G2	G2/A 091214	HCl-2xAS method	15	43	14,7	6,32	220,5	94,8
G2	G2/A 100911	HCl-2xAS method	15	41	21,5	8,82	322,5	132,3
G14	G14/A 081213	HCl-2xAS method	15	39	25,0	9,75	375	146,25
G14	G14/A 091214	HCl-2xAS method	15	40	16,5	6,60	247,5	99
G14	G14/A 100911	HCl-2xAS method	15	36	22,2	7,99	333	119,85
G18	G18/A 0912	HCl-2xAS method	15	42	15,0	6,30	225	94,5
G18	G18/A 100911	HCl-2xAS method	15	40	24,9	9,96	373,5	149,4
G2	G2/K 081213	Pierce Chicken IgY Purification Kit	17	82	6,5	5,33	110,5	90,61
G2	G2/K 091214	Pierce Chicken IgY Purification Kit	16	85	4,9	4,17	78,4	66,72
G2	G2/K 100911	Pierce Chicken IgY Purification Kit	15	84	5,7	4,79	85,5	71,85
G14	G14/K 081213	Pierce Chicken IgY Purification Kit	17	84	7,2	6,05	122,4	102,765
G14	G14/K 091214	Pierce Chicken IgY Purification Kit	16	86	5,1	4,39	81,6	70,24
G14	G14/K 100911	Pierce Chicken IgY Purification Kit	15	84	5,2	4,37	78	65,55
G18	G18/K 0912	Pierce Chicken IgY Purification Kit	16	83	5,0	4,15	80	66,4
G18	G18/K 100911	Pierce Chicken IgY Purification Kit	15	84	7,6	6,38	114	95,7
